# Food Insecurity Associated with Higher Stress, Depressive Symptoms, and Lower Diet Quality among Women Caregivers in North Carolina

**DOI:** 10.3390/nu16152491

**Published:** 2024-07-31

**Authors:** J. Lauren Butler, Cassandra M. Johnson, Annie Hardison-Moody, Sarah K. Bowen

**Affiliations:** 1 Nutrition and Foods Program, School of Family and Consumer Sciences, Texas State University, 601 University Drive, San Marcos, TX 78666, USA; laurenbutler@txstate.edu; 2 Nutrition and Dietetics Department, University of North Florida, Jacksonville, FL 32224, USA; 3 Department of Agricultural and Human Sciences, North Carolina University, Raleigh, NC 27695, USA; amhardis@ncsu.edu; 4 Department of Sociology and Anthropology, North Carolina University, Raleigh, NC 27695, USA; skbowen@ncsu.edu

**Keywords:** cross-sectional studies, young adult, middle aged, poverty, food supply, women’s health, mental health, rural

## Abstract

Background: Research suggests a bidirectional relationship between food insecurity and stress, but few studies have examined associations of food insecurity with stress and other indicators of cardiometabolic health, including depression, diet quality, and body weight, among lower-income women in the U.S. Methods: This cross-sectional study analyzed data from lower-income women caregivers living in North Carolina (n = 100): 42% Black/African American, 25% Hispanic/Latina, and 33% White women. Multivariable linear regression models were used to determine associations of food insecurity status with perceived stress, depressive symptoms, diet quality, and body mass index (BMI). Multivariable logistic regression models were used to determine associations of food insecurity with clinical depression and BMI ≥ 30 kg/m^2^. Associations were examined with and without adjustment for perceived stress. Results: Forty-two percent of the sample were experiencing food insecurity. Compared to food secure caregivers, food-insecure caregivers had significantly higher perceived stress (β: +7.51; 95%CI: 4.19, 10.84) and depressive symptoms (β: +3.55; 95%CI: 0.54, 6.56) and lower diet quality (β: −9.10; 95%CI: −15.81, −2.40). Associations with BMI outcomes were not statistically significant. Conclusion: Findings support removing stigma in nutrition assistance programs and clinical interactions, motivate future longitudinal studies, and inform the development of destigmatizing interventions for health promotion or disease prevention.

## 1. Introduction

The United States Department of Agriculture (USDA) defines food insecurity as a social and economic condition in which households experience “limited or uncertain availability of nutritionally adequate and safe foods, or limited or uncertain ability to acquire acceptable foods in socially acceptable ways [1]”. Food insecurity is part of a complex interplay of social determinants that influence nutrition and weight, such as poverty, racism, and trauma, including adverse childhood experiences [2,3]. Food insecurity has been associated with stress and depression [4], as well as suboptimal dietary intake of key nutrients and lower diet quality [5,6] in the United States (U.S.). Exposure to food insecurity may also increase the risk of adverse cardiometabolic health outcomes (e.g., cardiovascular disease and type 2 diabetes), including obesity, particularly for women [7,8,9].

The most recent report from the USDA Economic Research Service shows that in 2022, 12.8% of households in the U.S. had experienced food insecurity at some point in the past 12 months, meaning that roughly one out of every eight households dealt with food insecurity [10]. Rates of food insecurity are even higher among households with children (17.3%), households with children headed by a single woman (33.1%), households with non-Hispanic Black (22.4%) and Hispanic (20.8%) reference persons, and households with incomes below 100% of the federal poverty level (36.7%) [10]. Addressing food insecurity is a priority for federal agencies, including the USDA [11], and an important area of public health research and policy [12,13,14].

In a 2020 systematic review and meta-analysis, Pourmotabbed and colleagues indicated that food insecurity was associated with an increased risk of stress among women living in Canada, the U.S., and Mexico [4]. More recently, a cross-sectional study was conducted with lower-income women living with children who were eligible for the U.S. Special Supplemental Nutrition Program for Women, Infants, and Children [15]. Results showed the odds of mental distress increased concurrently with the severity of food insecurity [15]. Specifically, the odds of moderate to severe mental distress were 2.1 times higher among women with low food security than for women who were food secure [15]. Additional research on the relationship between stress and food insecurity among lower-income women with children is needed to build the evidence base.

Summarized evidence from longitudinal and cross-sectional studies indicates a cyclical or bidirectional relationship between stress and food insecurity [16,17,18]. Maynard et. al. proposed that chronic stress, including early childhood stress from adverse childhood experiences, may lead to economic instability, which then leads to food insecurity and stress [17]. Thus, stress may confound the associations of food insecurity with other cardiometabolic health indicators, like diet quality, that can be used to assess cardiometabolic disease risk [19]. Many studies have also examined how food insecurity is related to indicators of cardiometabolic health [8,20,21]. For example, a large body of research finds that food insecurity is associated with depression and poor diet quality. Findings are less consistent for food insecurity and risk of obesity [20]. Less is known about how the relationship of food insecurity with stress matters for depression, diet quality, and weight status. This question is especially important for lower-income women caregivers, who are at higher risk of food insecurity [17]. 

The primary aim of this study was to determine the association of food insecurity with stress in a sample of racially and ethnically diverse women caregivers from lower-income rural and urban communities in North Carolina. A secondary aim was to determine associations of food insecurity with stress-related cardiometabolic health indicators—depressive symptoms, diet quality, and weight status. A tertiary aim was to determine associations of food insecurity with clinical depression and obesity, two clinical endpoints that are relevant for cardiometabolic health. We hypothesized that participants experiencing food insecurity would report higher levels of perceived stress compared to those experiencing food security.

## 2. Methods

### 2.1. Study Design

This manuscript describes the results of a cross-sectional analysis of data from a mini-cohort study, The Voices into Action: The Families, Food, and Health study (VIA study), a community-based mixed-methods research and outreach project with lower-income rural and urban households in central North Carolina (NC). The study was completed between 2012 and 2020 [22]. The study aimed to understand the impacts of the food environment on family health and well-being, including food access, among lower-income families. Community outreach efforts focused on facilitating policy and environmental changes to increase access to healthy foods and places to be active.

**Figure 1:**
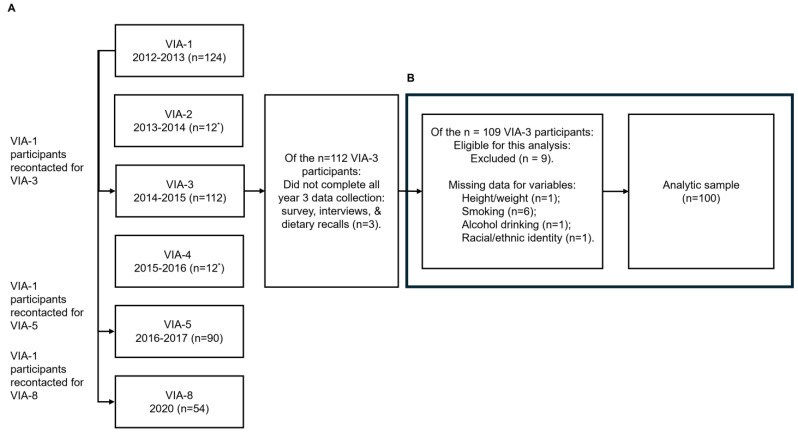
Flowchart for participants between enrollment at Year 1 and through Year 8 of the VIA study. This figure summarizes data collected across the entire VIA study, including details for the analytic sample used in this manuscript. The VIA study was a longitudinal mixed-methods research and outreach project. The number of participants in Year 1 is labeled as VIA-1. (**A**): Process to recontact VIA participants and engage them in continued data collection from baseline at Year 1 through Year 8. Sample size for the adult female participants is shown for each wave of data collection; exceptions are noted for sample size for households. (**B**): Process to create the analytic sample from Year 3 of the VIA study, including missing data. * Sample size is the number of participating households rather than the number of caregivers.

Women caregivers with an indexed child between 2 and 8 years of age at baseline were recruited and then contacted again to provide quantitative and qualitative data across multiple waves. Figure 1 shows a flowchart for years 1 through 8 of the study. Years 1, 3, and 5 included in-depth interviews, 24 h dietary recalls, surveys, and 24 h time diaries; Years 2 and 4 collected qualitative data with ethnographic or in-depth naturalistic observations over a 7-day period among a subset (about 10%) of households; Year 8 included in-depth interviews and surveys [22].

The VIA study received ethical approval from the Institutional Review Board at NC State University. Participants provided written informed consent before data collection. The study team, sample, and measures are described in more detail in the following section.

### 2.2. Study Team and Training

The VIA study team was interdisciplinary, composed of faculty members, graduate students, and staff with training and experience in public health, sociology, and nutrition and dietetics. Some team members had relevant lived experiences, such as being raised in lower-income households or rural communities or self-identifying as part of a racialized or marginalized population. Several team members were bilingual in English and Spanish.

Before starting data collection in Year 3 (2014–2015, Figure 1), researchers completed intensive training in data collection techniques, with demonstrations, discussions, and practice with protocols and instruments. Researchers who administered 24 h dietary recalls were trained in Nutrition Data System for Research (NDSR) research software versions 2012–2017, developed by the Nutrition Coordinating Center [University of Minnesota, Minneapolis, MN, retrieved from https://www.ncc.umn.edu/products/ (accessed on 30 June 2024)]. In addition, researchers completed training on how to train VIA study participants to use the NDSR Food Amount Booklet [23], and three-dimensional portion aids like measuring cups and spoons to report portion sizes for dietary recalls. All researchers were trained to obtain anthropometric data for children and adult females with digital scales and stadiometers using the standard National Health and Nutrition Examination Survey (NHANES) protocol for anthropometry [24]. The VIA study team met weekly to discuss data collection progress and challenges, and the dietary recall team held separate meetings to discuss dietary recalls and NDSR data entry. All VIA protocols and instruments were pre-tested before data collection. After data collection, VIA researchers completed quality control for data collection forms before data entry into an electronic database in Microsoft^®^ Access [Microsoft Corporation. (2014). Microsoft Access. Retrieved from https://office.microsoft.com/access, (accessed on 30 June 2024)].

### 2.3. Sample and Recruitment

To be eligible for the study, participants had to identify as women and be the primary caregiver of at least 1 child between ages 2 and 8. Eligible participants also had to have a household income at or below 200% of the federal poverty line at the start of the study. There were no eligibility criteria for the caregiver’s age. Participants were recruited from two rural counties (Harnett and Lee) and one urban community (Southeast Raleigh in Wake County) of North Carolina [22]. Black/African American, Hispanic/Latina, and White women participated in the study. The sample was designed to be representative of the racial and ethnic identities of the low and lower-income populations in the three study areas.

To recruit for data collection in Year 3, which is the focus of this manuscript, VIA researchers started community outreach in the spring of 2014 via mailed newsletters, phone calls, text messages, and emails to Year 1 study participants. Retention for Year 3 was 90%. For Year 3 data collection, VIA participants were compensated with USD 125 in cash. Participants received USD 25 for the first interview, USD 25 for the second interview, and USD 75 for the dietary recalls and survey at their last interview.

### 2.4. Measures

#### 2.4.1. Sociodemographic Characteristics

Each participant’s age, race or ethnicity, and highest level of education completed was collected at baseline or Year 1 using a background form that preceded an interviewer-administered survey. Age was calculated based on the participant’s reported birthdate and interview date. To obtain race or ethnicity data, participants were asked, “What do you consider your race or ethnicity to be?” For education, participants were queried on highest educational attainment: “No formal schooling”; “8th grade or less”; “Some high school (HS)”; “HS/GED”; “Trade/vocational school degree/certificate”, “Some college”, “Bachelor’s degree”, “Graduate/professional degree”. Finally, researchers also noted the caregiver’s relationship with the indexed child (i.e., mother or grandmother).

#### 2.4.2. Physical Activity

Because physical activity is associated with stress, previous studies have adjusted for physical activity when examining associations of weight-related outcomes [25,26]. Physical activity data were collected as part of an interviewer-administered survey. Participants reported the number of days (0–7) per week that they participated in at least 30 min of physical activity that made them breathe hard, or their heart beat faster than normal. For the current analyses, physical activity was defined based on the American Heart Association (AHA) recommendation for moderate-intensity aerobic activity [27], based on the 2nd edition of the Physical Activity Guidelines published by the U.S. Department of Health and Human Services [28]. The AHA defines moderate-intensity aerobic activity as an activity that makes one’s heart beat faster and makes one breathe harder than normal and recommends that adults engage in at least 150 min per week of moderate-intensity aerobic activity [27]. If participants reported participating in moderate-intensity activity for at least 30 min on 5 days a week, they would meet the AHA recommendations (30 min per day × 5 days per week = 150 min per week). Participants were categorized as reporting no aerobic physical activity (0 days per week), physical activity below recommended levels (1 day to 4 days per week), or physical activity at or above recommended levels (5+ days per week).

#### 2.4.3. Smoking and Alcoholic Beverage Consumption

Smoking and alcoholic beverage consumption were determined from the tobacco and alcohol section of the interviewer-administered survey. Participants were asked, “In the past 30 days, on how many days did you smoke at least one cigarette?”. Participants who reported smoking on at least one day were asked, “During the past 30 days, on the days that you smoked, how many cigarettes did you smoke per day on average?”. Participants who answered “none” to smoking in the past 30 days were categorized as non-smokers. Those who reported smoking at least one cigarette in the past 30 days were categorized as smokers and further categorized based on the number of cigarettes smoked per day. Smoking status was defined as a non-smoker, smoker with less than half a pack of cigarettes (<10), or smoker with half a pack or more of cigarettes (10+) per day [29,30]. Participants were asked, “In the past 30 days, on how many days did you drink any type of alcoholic beverage?”. Participants who reported drinking alcohol on at least one day were asked, “When you drank, how many drinks did you have per day? A “drink” is a glass of wine, a can or bottle of beer, a wine cooler, a shot glass of liquor, or a mixed drink”. For the current study, drinking status was defined based on the U.S. Centers for Disease Control and Prevention’s (CDC) definition of moderate drinking as no more than 1 drink per day for women [31]. Those who reported drinking no alcoholic drinks in the past 30 days were categorized as non-drinkers. Those who reported drinking at least one drink in the past month were categorized based on the number of alcoholic drinks per day as either a drinker with no more than 1 drink per day, or a drinker exceeding 1 drink per day.

#### 2.4.4. Total Energy Intake and Diet Quality

To obtain total energy intake and diet quality data, trained researchers administered 24 h dietary recalls over the phone using the multiple-pass method based on the NHANES protocol for dietary interviews [32]. Each participant completed at least two 24 h dietary recalls on non-consecutive days, including one weekday and one weekend day, over a two-week period. Participants reported amounts using printed versions of the NDSR Food Amount Booklet [23], CDC handout [33], and actual measures of participants’ commonly used bowls, cups, and spoons. VIA researchers measured the volume of commonly used household items with dry beans during dietary recall training. Dietary intake data were entered into NDSR for nutrient analysis and used to generate NDSR output files (University of Minnesota, Nutrition Coordinating Center. Research software versions 2012–2017). 

Total energy intake was calculated as the average per-person total energy intake. Diet quality was assessed using the Healthy Eating Index 2010 total score, HEI-2010. At the time of data collection, the HEI-2010 was a reliable and valid measure of overall diet quality for the U.S. population aged 2 years and older, including pregnant and breastfeeding women and those with varied dietary patterns [34,35]. The HEI-2010 total score ranges from 0 to 100; higher scores represent greater adherence to the recommendations in the 2010 Dietary Guidelines for Americans [36]. Individual age and activity level and average values of dietary intake variables, such as total energy, were used to calculate the HEI-2010 scores. The data analysis to create HEI-2010 scores was generated using SAS software, version 9.4 of the SAS System for Mac OS X Copyright © 2013 SAS Institute Inc. SAS and all other SAS Institute Inc. product or service names are registered trademarks or trademarks of SAS Institute Inc., Cary, NC, USA [https://www.sas.com/en_us/legal/editorial-guidelines.html (accessed on 30 June 2024)]. Websites, including SAS scoring macros, were used to calculate HEI-2010 scores from NDSR output files [37,38,39,40].

#### 2.4.5. Weight, Height, and Body Mass Index

During in-person nutrition interviews, trained researchers used the NHANES protocol for anthropometry [24] to obtain measured weight in pounds and height in inches with a digital scale and a portable stadiometer. Body mass index (BMI) was calculated based on the formula weight (kg)/[height (m)]^2^ using average weight (kg) and average height (m). Recommended cut-offs were used to categorize BMI for weight status [41]. Participants also were categorized as living with obesity (BMI ≥ 30 kg/m^2^) or not living with obesity (BMI < 30 kg/m^2^).

#### 2.4.6. Food Insecurity

An interviewer-administered the 10-item version of the U.S. Food Security Survey Module (FSSM) in order to assess adult-level food insecurity over the past 30 days [42]. Specifically, participants were asked to respond with (1) often true, (2) sometimes true, or (3) never true to food-related statements, and (1) yes or (2) no to food-related questions. For example, participants were asked to respond to the following statement “(I/We) worried whether (my/our) food would run out before (I/we) got money to buy more.” and to answer the following question, “In the last 30 days, did (you/you or other adults in your household) ever cut the size of your meals or skip meals because there wasn’t enough money for food?” [42]. Per FSSM guidelines, food insecurity status was coded into binary categories based on the total number of affirmative responses to the FSSM. Participants with 0–2 affirmative responses were coded as experiencing food security, and those with 3–10 affirmative responses were coded as experiencing food insecurity [42].

#### 2.4.7. Stress and Depression

An interviewer administered surveys to measure stress and depression. Perceived stress was measured using Cohen’s Perceived Stress Scale (PSS), a 10-item scale [43,44]. The PSS is a valid, reliable measure of perceived or appraised stress among U.S. adults [43,44]. The PSS-10 scores range from 0 to 40, with scores of 14 to 26 typically regarded as moderate perceived stress [43,45]. Depressive symptoms were assessed with 20 items from the Center for Epidemiological Studies Depression Scale Revised (CESD-R), a valid and reliable measure for U.S. adults [46,47,48]. CESD-R scores range from 0 for those who say “not at all or less than one day” to all 20 questions and 60 for those who say “5–7 days“ or “nearly every day for 2 weeks” for all 20 questions [47]. Clinical depression was defined using the Center for Epidemiological Studies depressive symptom algorithm for the CESD-R [47]. Participants were categorized as living with clinically significant depression (score ≥ 16) or not living with depression (score < 16) [48,49].

### 2.5. Statistical Analysis

In terms of sample size, this study analyzed secondary data from participants in Year 3 of the VIA study, which did not include an a priori sample size calculation for testing associations. This analysis included all participants who completed interviews, dietary recalls, and surveys in Year 3 (n = 109). Data from participants who were missing data for the main exposure or outcomes (n = 1 missing BMI) or covariates: education (n =1), smoking (n = 6), or alcohol (n = 1) were excluded. Due to the small sample size, participants who reported “mixed or other” race or ethnicity were excluded from this analysis (n = 1). The final analytic sample included data from 100 participants (Figure 1).

All data analyses were conducted using Stata, version 17 (StataCorp, College Station, TX, USA). All variables were defined, and distributions of variables were examined. Descriptive statistics were used to assess central tendency. For continuous variables, means and standard deviations were calculated; for categorical variables, percentages were calculated. To test for differences by food insecurity status, chi square tests were used to determine differences in the distribution of categorical covariates and analysis of variance (ANOVA) was used to test means of continuous covariates. Multivariable linear regression models were used to examine associations of food security status with perceived stress, depressive symptoms, diet quality, and body mass index (BMI). Outcomes (continuous) were regressed on food security status coded as a binary variable (0/1). To examine associations of food security status with clinical depression and obesity, multivariable logistic regression models were used to regress binary outcomes (0/1) on a binary measure of food security status (0/1). “Food secure” was the referent group in all models.

All models were adjusted for the following covariates: age (continuous), race/ethnicity (Black/African American, Hispanic/Latina, White), birthplace (U.S., Outside the U.S.), educational attainment (less than high school (HS), HS diploma or GED, HS graduate or postsecondary education), physical activity (none, 1–4 days/week, ≥5 days/week), smoking (none, <10 cigarettes/day, ≥10 cigarettes/day), and alcohol (none, ≤1 drink/day, >1 drink/day). Adjustment variables were determined based on a similar study of food insecurity and diet quality for lower-income adults in the U.S. by Leung and colleagues [25]. Previous studies also suggested controlling for physical activity in research with stress [25,26]. Alcohol was included as an adjustment variable given prior research on alcohol with weight status and dietary intake [50,51]. Previous research suggests controlling for total energy intake when modeling BMI categories as an outcome [50]. Thus, for models with BMI as an outcome, total energy intake was included. Associations of food security with depression, diet quality, and BMI were examined with and without adjustment for stress.

## 3. Results

### 3.1. Sample Characteristics

The analytic sample was made up of 42% Black/African American, 25% Hispanic/Latina women, and 33% White participants. The average age of participants was 36.9 ± 9.8 years (Table 1). All participants were mothers (n = 90) or grandmothers (n = 10). Forty-two percent of participants were categorized as having experienced food insecurity in the 30 days prior to the interview [42]. The mean total sample perceived stress score corresponds to moderate perceived stress (15.2 ± 8.0), whereas the population average for depressive symptoms was consistent with clinically significant depression (19.1 ± 7.0). Food-insecure caregivers had higher perceived stress scores (19.2 ± 7.2 vs. 12.3 ±7.4; *p* ≤ 0.001), higher depressive symptom scores (21.0 ± 8.15 vs. 17.8 ± 7.8; *p* = 0.02), and lower diet quality scores (46.8 ± 16.7 vs. 54.4 ± 17.3; *p* = 0.03) than food-secure caregivers.

### 3.2. Associations of Food Insecurity with Perceived Stress, Depressive Symptoms

Unadjusted and adjusted differences in stress and depressive symptoms by food security status are presented in Table 2. Compared to women caregivers experiencing food security, those experiencing food insecurity had statistically significantly higher total stress (β: +7.51; 95% CI: 4.19, 10.84) and depressive symptoms (β: +3.55; 95% CI: 0.54, 6.56) (Model 1).

### 3.3. Associations of Food Insecurity with Diet Quality and Weight Status

Unadjusted and adjusted associations of food security with diet quality and weight status are presented in Table 3. The diet quality of food-insecure caregivers (β: −7.58; 95% CI: −14.44, −0.72) was significantly lower than that of food-secure caregivers. The association was strengthened in adjusted Model 1 (β: −8.96; 95% CI: −14.66, −3.27). After adjusting for stress in Model 2, the association was strengthened (β: −9.70; 95% CI: −16.06, −3.34) (Model 2). Food security status was not statistically significantly associated with BMI. However, the association was positive in the unadjusted model and adjusted Model 1, whereas the association was negative after adjustment for stress in Model 2.

### 3.4. Associations of Food Insecurity with Clinical Depression and Obesity

Food insecurity was not statistically significantly associated with odds of clinical depression or obesity (Table 4). In Model 1, those experiencing food insecurity had higher odds of clinical depression (OR: 1.42; 95% CI: 0.50, 4.01). After adjustment for stress in Model 2, the odds of clinical depression (OR: 0.42; 95% CI: 0.11, 1.65) were lower for those experiencing food insecurity. The odds of obesity were lower among food-insecure caregivers compared to food-secure caregivers, and the association was attenuated after adjustment for covariates and stress.

## 4. Discussion

In this mini-cohort study of women caregivers living in lower-income rural and urban communities in North Carolina, food insecurity was associated with higher stress and depressive symptoms and lower diet quality. Recent research has shown food insecurity to be a powerful health determinant [7,52]. This study’s findings underscore the need to address food insecurity and stress together in research, practice, and policy.

These findings indicate that food insecurity is associated with increased stress, in line with other cross-sectional studies of food insecurity, stress [4,20], and mental health among women in high-income countries [17]. A global meta-analysis of food insecurity examined data from 10 countries across three continents: North America, Europe, and Asia [4]. The study found that associations of food insecurity with adverse mental health and psychosocial stressors held for different regions even after adjustment for confounding variables, although households in North America had the highest risk of stress and anxiety compared to other regions (e.g., Asia and Europe) [4]. Our findings bolster the importance of understanding how food insecurity may contribute to higher stress and adverse health outcomes for women living in high-income countries [4,17].

Given the cross-sectional nature of this study, we are unable to determine the pathways that link stress and food insecurity. Previous studies have suggested pathophysiology linking food insecurity to cardiometabolic diseases through three different pathways: (1) behavioral, where food insecurity results in suboptimal diet quality and increases metabolic risk; (2) stress, where food insecurity “forces tradeoffs” in meeting basic needs and limits chronic disease self-management or treatment; and (3) inflammation, where food insecurity disrupts eating patterns and increases systemic inflammation or where food insecurity reduces diet quality changes gut microbiota and increases risk of cardiometabolic disease [53]. Other research on food insecurity and adverse cardiometabolic health outcomes supports these pathways [7,8]. Previous research has examined the role of stress in the association of food insecurity with dietary intake of hyperpalatable foods and indicators of cardiometabolic disease among Black and White women in the U.S. (36–43 years old) and found that cortisol intensified the association of food insecurity on blood glucose levels [54]. They emphasized that the stress related to food insecurity may contribute to poor mental health and increase the risk of depression [54]. Related research on adverse childhood experiences (ACEs) and cardiometabolic health aids the understanding of the negative impacts of food insecurity and stress on cardiometabolic health outcomes [55]. For example, a meta-analysis identified social disruption, changes in health behaviors (e.g., eating, exercise), chronic stress response, including the role of weight stigma, and mental health (e.g., depression) as explanations for the negative health impact of ACEs [55].

This study also found that food insecurity was associated with higher depressive symptoms and elevated odds of clinical depression, supporting other studies [4,17,20,56]. However, estimates for clinically significant depression were not statistically significant. Our inability to account for life phases and circumstances may contribute to the lack of statistical significance in our study findings. In a 2018 study of the Twin Cities Healthy Start Program data, the odds of having elevated depressive symptoms were statistically significantly higher for women in the prenatal period with moderate or high levels of food insecurity compared with those with low levels of food insecurity [57]. Elevated odds were not statistically significant for women in the postpartum period [57]. Further, in a cross-sectional study of mostly non-Hispanic White women born in the U.S. who did not have children, Loh et al. reported statistically significantly higher odds of depression among food-insecure participants [49]. However, estimates were not statistically significantly different for those with low food security who were participating in a food and nutrition assistance program (the Special Supplemental Nutrition Program for Women, Infants, and Children or WIC) [49]. Furthermore, after adjusting for stress, we saw a change in estimate direction. The odds of clinical depression were lower for women experiencing food insecurity. Longitudinal studies with larger samples are needed to investigate whether stress is a strong confounder or a mediator of the association between food insecurity and depression.

In addition, this study showed that food insecurity was associated with lower diet quality for women caregivers. There are different potential explanations for why food insecurity is correlated with lower diet quality [7]. People may use management and coping strategies to avoid or mitigate food insecurity within their households, and the “tradeoffs” in food choices may reduce diet quality. For example, obtaining more energy (or kilocalories) from lower-priced foods that are more filling negatively influences diet quality. Additionally, people may also use food or beverages (e.g., coffee or soda) as comfort and engage in emotional eating or drinking in response to trauma [58] or food insecurity [59]. Another possibility is that increased stress and depression may lead to dietary intake of highly palatable foods that are typically higher in fat, salt, and sugar and lower in quality [54,60]. Thus, the association of food insecurity with diet quality may be mediated by stress or depression.

Our study did not find associations with BMI outcomes (e.g., BMI as a continuous variable and BMI > 30 kg/m^2^). Untangling the relationship between obesity and food insecurity is complex, and existing research demonstrates conflicting results [21]. Previous studies show differential associations of food insecurity with obesity risk according to racial or ethnic identity among women in the U.S. [8,61,62]. Specifically, cross-sectional studies have found similar results for this association among non-Hispanic Black women [63,64]. Given that 42% of this study’s sample was Black/African American, our estimates may be most reflective of the association of food insecurity with weight status among Black/African American women in the U.S.

There are several implications of this study. First, findings point to the need to understand and eliminate the stigma that is associated with many food and nutrition assistance programs [65,66,67], including the Supplemental Nutrition Assistance Program. Other scholars have introduced the concept of intersectional stigma—the convergence of people living with multiple marginalized or stigmatized identities based on race, class, gender, and other social categories—and its effects on individual behavior and physical and mental health outcomes [66]. Given that stigma increases stress and erodes mental health [66], applying intersectional approaches that prioritize stigma reduction will help advance policy changes for nutrition and food assistance programs. Leading scholars have advocated for a right-to-food approach, which can inform policy, systems, and environmental changes to address food insecurity [68,69]. Second, in terms of practice, these findings indicate that food insecurity should be assessed in clinical settings as a measure of social risk, as recommended by others in health care [70]. Third, more broadly, these findings justify training for students in health professions (e.g., nutrition and dietetics, medicine) regarding the determinants, experiences, and consequences of food insecurity and stigma. Given that food insecurity contributes to poorer diet quality and mental health outcomes, it is important for health practitioners to understand food insecurity and be able to make referrals to help prevent and alleviate it [68,69]. Likewise, health practitioners need to avoid stigma in interactions with clients or patients and consider trauma-informed practices to avoid increasing stress [71,72].

Regarding limitations, this study is limited by a small sample size (n = 100), which constrained data analysis. For example, food insecurity was categorized as a binary variable rather than a three- or four-level variable because there were small cell sizes for some adjustment variables, such as education. Another limitation is that the study did not compare food insecurity or nutrition-related outcomes between urban and rural residents, even though previous research suggests that experiences of food insecurity may differ considerably in rural and urban places [73]. In addition, observational studies like this one are unable to control completely for confounding bias, which obscures the association between exposure and outcome. Finally, the authors acknowledge the limitations of using BMI as a measure of body composition or as a clinical diagnostic tool. In the current study, BMI was used for sample-level categorization and not as an individual-level diagnostic indicator of health status.

The strengths of this study are related to the quality of the data. The VIA study employed several strategies for reliability and validity, including thoroughly training researchers, creating standardized instruments for data collection, and establishing clear protocols for data entry. Strategies for validity included working with an experienced and interdisciplinary group of team members, selecting strong and validated measures for the main exposure and outcomes, using recommended NHANES protocols for dietary assessment and anthropometry, engaging in peer debriefing during data collection and management to address and resolve challenges, and employing rigorous processes for quality control of data entry.

## 5. Conclusions

This study’s findings underscore the importance of designing food and nutrition assistance programs to reduce stigma. The results support people-centered policy actions that enhance autonomy and support the mental health and well-being of women participating in the Supplemental Nutrition Assistance Program or other food and nutrition assistance programs. Practice implications include integrating measures of food insecurity and mental health into clinical visits. Health practitioners can facilitate connections between people experiencing food insecurity and community-based resources for food and mental health. Toward that goal, this study’s findings support training health profession students so they can understand and identify food insecurity as they work to prevent or mitigate it. Health professionals’ interactions must aim to reduce stigma, which will avoid increased stress.

Future research should use longitudinal designs to further examine the relationships between food insecurity, stress, and depression as part of understanding cardiometabolic health outcomes. Specifically, longitudinal studies that explore interactions between stress and dietary behaviors are needed. At the same time, future research might examine how life-course factors (e.g., pregnancy) and participation in food and nutrition assistance programs shape the relationships between food insecurity and mental health. Additional research is warranted to understand the role of stress in differential associations of food insecurity across BMI categories and racial and ethnic groups. Future studies might explore new interventions that mitigate food insecurity without shame or stigma as part of health promotion and disease prevention. A critical examination of the public health discourse around complex health conditions, especially obesity, is essential for addressing food insecurity in equitable ways. Ultimately, researchers can work with communities to design, implement, and evaluate culturally relevant and destigmatizing interventions related to food insecurity.

## Figures and Tables

**Table 1:** Characteristics of women caregivers in Year 3 of the VIA study by food insecurity status ^a^.

Characteristics	Total	Participants Experiencing Food Security	Participants Experiencing Food Insecurity	*p*
n (%)	100 (100%)	58 (58%)	42 (42%)	0.1082
Age, years (mean ± SD)	36.9 ± 9.8	35.4 ± 9.3	39.1 ± 10.1	0.0612
Race or Ethnicity [N (%)]				0.007
White	33 (33%)	12 (20.7%)	21 (50%) ^j^	
Black/African American	42 (42%)	30 (51.7%)	12 (28.6%)	
Latina/Hispanic	25 (25%)	16 (27.6%)	9 (21.4%)	
Birthplace [N (%)]				0.544
U.S.	78 (78%)	44 (75.9%)	34 (81%)	
Outside of U.S.	22 (22%)	14 (24.1%)	8 (19.1%)	
Education [N (%)]				0.426
<High school	31 (31%)	15 (25.9%)	16 (38.1%)	
High school	29 (29%)	18 (31%)	11 (26.2%)	
>Highschool	40 (40%)	25 (43.1%)	15 (35.7%)	
Physical Activity [N (%)] ^b^				0.791
None	60 (60%)	16 (27.6%)	28.6%)	
1–4 days/week	23 (23%)	23 (39.7%)	14 (33.3%)	
≥5 days/week	17 (17%)	19 (32.8%)	16 (38.1%)	
Smoking [N (%)] ^c^				0.198
None	61 (61%)	35 (60.3%)	25 (59.5%)	
<10 cigarettes/day	18 (18%)	16 (27.6%)	7 (16.7%)	
≥10 cigarettes/day	21 (21%)	7 (12.1%)	10 (23.8%)	
Alcohol [N (%)] ^d^				0.957
None	17 (17%)	35 (60.3%)	26 (61.9%)	
≤1 drink/day	25 (25%)	11 (19%)	7 (16.7%)	
>1 drink/day	58 (58%)	12 (20.7%)	9 (21.4%)	
Total Energy Intake, kcal/day (mean ± SD) ^e^	1755.3 ± 531.4	1726.9 ± 475.8	1794.9 ± 467	0.5286
Diet quality (mean ± SD) ^f^	51.2 ± 17.4	54.4 ± 17.3	46.8 ± 16.7	0.0307
BMI, kg/m^2^ (mean ± SD) ^g^	33.4 ± 8.8	33.1 ± 8.8	33.7 ± 8.8	0.7457
BMI Category kg/m^2^ [N (%)]				0.973
18.5 to 24.9	17 (17%)	10 (17.2%)	7 (16.7%)	
25.0 to 29.9	25 (25%)	14 (24.1%)	11 (26.2%)	
≥30.0	58 (58%)	34 (58.6%)	24 (57.1%)	
Depressive Symptoms (mean ± SD) ^h^	19.1 ± 7.0	17.8 ± 5.8	21 ± 8.2	0.0243
Depression [N (%)] ^h^				0.973
No clinical depression	33 (33%)	21 (36.2%)	12 (28.6%)	
Clinically significant depression	67 (67%)	37 (63.8%)	30 (71.4%)	
Stress (mean ± SD) ^i^	15.2 ± 8.0	12.3 ± 7.4	19.2 ± 7.2	0.0000
FSSM Affirmative Responses (mean ± SD)	2.5 ± 2.5	0.62 ± 0.77	5.07 ± 1.76	0.0000

^a^ Data for 100 participants included in the analytic sample of participants in Year 3 of the VIA study (2014–2015). Food insecurity status was defined using the Food Security Survey Module (FSSM) scores as individuals experiencing food security (“food secure”, raw score 0–2) or food insecurity (“food insecure”, raw score 3–10). Sociodemographic and behavioral characteristics and nutrition-related outcomes assessed with surveys, dietary recalls, and anthropometry. Values are means ± standard deviation (SD) for continuous covariates and percentages for categorical covariates. *p*-values for chi^2^ tests of the unadjusted percentage distributions of categorical covariates and uncorrected overall *p*-value for analysis of variance (ANOVA) for means of continuous covariates. ^b^ Categorized according to the American Heart Association’s recommendations for participating in moderate-intensity cardiovascular activity for at least 30 min per day for 5 days a week. ^c^ Categorized according to the U.S. Code of Federal Regulations Title 21 minimum cigarette pack size. ^d^ Categorized according to the U.S. Centers for Disease Control and Prevention definition of moderate drinking as no more than 1 drink per day for women. ^e^ Total energy intake in kilocalories (kcal) per day was averaged across 24 h dietary recalls. ^f^ Diet quality was assessed using the Health Eating Index (HEI) 2010 total score based on 24 h dietary recalls. ^g^ Body Mass Index (BMI) based on measured height in meters (m) and weight in kilograms (kg). ^h^ Depressive symptoms were assessed using the Center for Epidemiological Studies Depression Scale Revised (CESD-R). Participants were categorized as living with clinically significant depression (CESD-R score ≥ 16) or not living with depression (CESD-R score < 16). ^i^ Perceived stress was measured using Cohen’s Perceived Stress Scale. ^j^ Different than food secure group (*p* = 0.007).

**Table 2:** Predicted beta coefficients and 95% confidence intervals (CI) describing differences in stress and depressive symptoms between women caregivers experiencing food security and food insecurity in Year 3 of the VIA study ^a^.

	Unadjusted			Model 1 ^b^		
A: Stress	β	95% CI	Beta	β	95% CI	Beta
Food Secure	Ref			Ref		
Food Insecure	6.90	3.96, 9.84	0.43	7.51	4.19, 10.84	0.46
	**Unadjusted**			**Model 1 ^b^**		
**B: Depressive Symptoms**	**β**	**95% CI**	**Beta**	**β**	**95% CI**	**Beta**
Food Secure	Ref			Ref		
Food Insecure	3.18	0.27, 6.09	0.23	3.55	0.54, 6.56	0.25

^a^ Data for 100 participants included in the analytic sample in Year 3 of the VIA study (2014–2015). Values are beta coefficients (β) and 95% confidence intervals (95% CI) and standardized beta coefficients (Beta) obtained from multivariable linear regression models. Food insecurity was defined using the Food Security Survey Module scores as food security (raw score 0–2) or food insecurity (raw score 3–10). Continuous outcomes vary for each regression model as follows: A: Stress defined using Cohen’s Perceived Stress Scale total score; B: Depressive symptoms defined using the Center for Epidemiological Studies Depression Scale Revised total score. All estimates are differences in stress or depressive symptom scores as compared to those experiencing food security. ^b^ Model 1 was adjusted for age, race or ethnicity, education, physical activity, smoking, and alcohol.

**Table 3:** Predicted beta coefficients and 95% confidence intervals (CI) describing the differences in diet quality and weight status between women caregivers living with food security and food insecurity in Year 3 of the VIA study ^a^.

	Unadjusted			Model 1 ^b^			Model 2 ^c^		
A: Diet Quality	β	95% CI	Beta	β	95% CI	Beta	β	95% CI	Beta
Food secure	Ref			Ref					
Food insecure	−7.58	−14.45, −0.72	−0.22	−8.96	−14.66, −3.27	−0.26	−9.70	−16.06, −3.34	−0.28
	**Unadjusted**			**Model 3 ^d^**			**Model 4 ^e^**		
**B: Weight Status**	**β**	**95% CI**	**Beta**	**β**	**95% CI**	**Beta**	**β**	**95% CI**	**Beta**
Food secure	Ref			Ref			Ref		
Food insecure	0.58	−2.95, 4.11	0.03	0.07	−3.78, 3.91	0.00	−0.27	−4.59, 4.05	−0.02

^a^ Data for 100 participants included in the analytic sample from Year 3 of the VIA study (2014–2015). Values are beta coefficients (β) and 95% confidence intervals (95% CI) and standardized beta coefficients (Beta) obtained from multivariable linear regression models. Food insecurity was defined using the Food Security Survey Module scores as food security (raw score 0–2) or food insecurity (raw score 3–10). Continuous outcomes vary for each regression model as follows: A: Diet quality using the Health Eating Index-2010 total score; B: Weight status using Body Mass Index. All estimates are the difference in diet quality or weight status as compared to those living with food insecurity. ^b^ Model 1 was adjusted for age, race or ethnicity, education, physical activity, smoking, and drinking status. ^c^ Model 2 was adjusted for Model 1 covariates plus Perceived Stress Scale (PSS) score (continuous). ^d^ Model 3 was adjusted for adjusted for Model 1 covariates plus total energy intake (continuous). ^e^ Model 4 was adjusted for adjusted for Model 3 covariates plus PSS score (continuous).

**Table 4:** Odds ratios (OR) and 95% confidence intervals (CI) of living with depression or obesity among women caregivers experiencing food security compared to food insecurity in Year 3 of the VIA study ^a^.

	Unadjusted		Model 1 ^b^		Model 2 ^c^	
A: Clinical Depression	OR	95% CI	OR	95% CI	OR	95% CI
Food Secure	1.00		1.00		1.00	
Food Insecure	1.42	0.60, 3.36	1.42	0.50, 4.01	0.42	0.11, 1.65
	**Unadjusted**		**Model 3 ^d^**		**Model 4 ^e^**	
**B: Obesity**	**OR**	**95% CI**	**OR**	**95% CI**	**OR**	**95% CI**
Food Secure	1.00		1.00		1.00	
Food Insecure	0.94	0.42, 2.11	0.73	0.26, 2.05	0.67	0.22, 2.07

^a^ Data for 100 participants included in the analytic sample at Year 3 (2014–2015). Values are odds ratios (OR) and 95% confidence intervals (95% CI) obtained from multivariable logistic regression models. Food insecurity was defined using the Food Security Survey Module scores as food security (raw score 0–2) or food insecurity (raw score 3–10). Binary outcomes vary for each regression model as follows: A: Clinical depression was defined as scoring >16 on the Center for Epidemiological Studies Depression Scale Revised (0/1); B: Obesity was defined as living with a Body Mass Index ≥ 30 kg/m^2^ (0/1). ^b^ Model 1 was adjusted for age, race or ethnicity, education, physical activity, smoking, and alcohol. ^c^ Model 2 was adjusted for Model 1 covariates plus Perceived Stress Scale score (continuous). ^d^ Model 3 was adjusted for adjusted for Model 1 covariates plus total energy intake (continuous). ^e^ Model 4 was adjusted for adjusted for Model 3 covariates plus PSS score (continuous).

## Data Availability

Restrictions apply to the availability of these data. Data were obtained from the Principal Investigators of the Voices into Action: The Families, Food, and Health (VIA) study through acceptance as a research affiliate, a data use agreement, and an accepted proposal for the analysis presented. Please email Sarah K. Bowen or Annie Hardison-Moody to inquire about opportunities as a research affiliate.
